# Rituximab induces phenotypical and functional changes of NK cells in a non-malignant experimental setting

**DOI:** 10.1186/s13075-016-1101-3

**Published:** 2016-09-15

**Authors:** Wolfgang Merkt, Hanns-Martin Lorenz, Carsten Watzl

**Affiliations:** 1 Department of Hematology, Oncology and Rheumatology, Internal Medicine V, University Hospital of Heidelberg, Heidelberg, Germany; 2 Leibniz Research Center for Working Environment and Human Factors at TU Dortmund (IfADo), Dortmund, Germany

**Keywords:** Natural killer cells, Rituximab, B cell depletion, Rheumatic diseases, CD137/41BB, Vasculitis, Granuloma

## Abstract

**Background:**

Rituximab has broad and increasing application in rheumatic diseases. It is known from lymphoma studies that natural killer (NK) cells can lyse rituximab-coated transformed B cells. However, the role of NK cells in mediating rituximab-induced depletion of non-malignant B cells is unknown. The purpose of this study was to provide fundamental data on rituximab-mediated effects on NK cells in PBMCs without tumor cells, in order to simulate effects that could be relevant in patients with rheumatic disease.

**Methods:**

Freshly isolated peripheral blood mononuclear cells (PBMCs) from healthy donors were cultured overnight with therapeutic antibodies. NK cells were isolated using a commercial kit or depleted from PBMCs using anti-CD56 and anti-CD16 monoclonal antibodies and magnetic beads. Cells were analyzed by multicolor flow cytometry. Cytotoxicity assays were performed using ^51^Cr-labeled K562 target cells.

**Results:**

Addition of rituximab to PBMCs resulted in depletion of B cells, which was dependent on NK cells and serum factors. The extent of B cell depletion correlated with the percentage of NK cells. Following incubation with rituximab, NK cells within PBMCs were activated, degranulated and downregulated the low affinitiy Fc-γ-receptor CD16 (FcγRIIIA). The co-activating receptor CD137 (41BB) was upregulated on a fraction of NK cells. NK cell function was altered in some donors in whom we observed rituximab-dependent reduction in NK cell cytotoxicity towards K562 tumor cells.

**Conclusions:**

NK cells mediate rituximab-induced B cell depletion. Rituximab induces altered NK cell phenotype and function.

**Electronic supplementary material:**

The online version of this article (doi:10.1186/s13075-016-1101-3) contains supplementary material, which is available to authorized users.

## Background

Natural killer (NK) cells are tightly regulated lymphocytes with cytotoxic activity against stressed and/or antibody-coated cells [1, 2]. The majority of human peripheral blood NK cells are CD56^dim^ NK cells bearing the low affinity Fc-γ-receptor CD16 (FcγRIIIA). CD16 binds IgG1 and mediates antibody-dependent cellular cytotoxicity (ADCC) [3]. CD16 plays a prominent role in activating NK cells [4]. Following stimulation, NK cells downregulate CD16 [5], by means of shedding [6] and intracellular uptake [7]. CD137 (41BB) is a co-activating receptor on NK cells that is upregulated upon binding of certain antibodies to CD16 [8, 9], but a possible upregulation upon binding of rituximab has not yet been reported.

Rituximab is a chimeric antibody that targets CD20 present on healthy and malignant B cells, and mediates depletion of these cells in vivo. It is used as a therapeutic agent in a large range of malignant and autoimmune diseases. Potentially, rituximab can trigger three effector functions [10]: (1) programmed cell death, (2) induction of complement-mediated cytotoxicity, and (3) ADCC mediated by Fc-γ-receptor-bearing immune cells, including CD16 on NK cells.

As shown in mouse models, Fc-γ-receptor-dependent mechanisms contribute substantially to the action of rituximab [11]. Clinical studies in humans confirm the important role of CD16 and NK cells in rituximab-mediated effects [12–17]. NK cells are activated in patients with lymphoma after rituximab infusion [18]. In addition, in vitro studies describe specific interactions between rituximab-coated (tumor) cells and NK cells [19, 20]. These data establish that NK cells are important mediators of rituximab-induced tumor cell lysis. In line with this, combination of rituximab and other immunotherapies are newly explored to enhance NK cell cytotoxicity [21].

In rheumatology, rituximab has numerous on-label and off-label applications, such as in rheumatoid arthritis and granulomatosis with polyangiitis (GPA) [22]. In these settings, elimination of autoimmune or over-activated non-malignant B cells is believed to mediate its therapeutic effect. Accordingly, rituximab is also referred to as B cell therapy. Nevertheless, B cell depletion in peripheral blood can be incomplete [23] and treatment response frequently fails [24] or is incomplete or delayed. The reasons for this limited efficacy are unknown and there are no predictors to identify patients who will not benefit from rituximab.

Importantly, most studies investigating the mechanisms of action of rituximab were performed in tumor settings. Knowledge about rituximab-induced alterations of NK cells in non-malignant contexts is very limited. One study describes the modification of rituximab-induced ADCC by recognition of additional tumor-specific NK cell receptor ligands [19]. Therefore, the mechanisms of rituximab-induced tumor cell lysis may not completely apply for the elimination of non-malignant B cells, and investigation of rituximab-mediated effects in non-malignant settings is needed in order to better understand and guide rituximab therapy in rheumatic disease.

Finally, we hypothesized that in addition to the “primary” B cell depletion, rituximab could exert secondary effects on NK cells via Fc-CD16-interactions. In line with this, Capuano et al. recently reported that pretreatment with rituximab-coated tumor cells leads to general inhibition of NK cell cytotoxicity [7]. It is not known whether NK cells that have been exposed to rituximab have altered functions in a non-malignant context.

The purpose of this study is to provide fundamental data on rituximab-mediated effects on NK cells in peripheral blood mononuclear cells (PBMCs) without tumor cells, in order to simulate effects that could be relevant in patients with rheumatic disease.

## Methods

### Donor consent and ethical approval

Informed consent was obtained from the healthy donors before donation of blood. The ethics committee of the University of Heidelberg approved this study.

### PBMC and NK cell preparation

PBMCs were isolated by density gradient centrifugation using Pancoll medium (PAN-Biotech GmbH, Aidenbach, Germany) according to the manufacturer’s recommendations. PBMCs were used freshly whenever possible or frozen on the day of blood donation. The anti-CD20 IgG1 antibody rituximab was used in saturated concentrations (10 μg/ml). Anti-tumor necrosis factor (TNF) alpha monoclonal IgG1 antibody infliximab and intravenous immunoglobulins (IvIgs) were used as controls (10 μg/ml). All three agents can theoretically bind CD16 and are used as therapeutic agents in rheumatic disease. IvIg had been shown to induce phenotypical and functional changes in NK cells when applied in high doses (10 mg/ml) [25]. NK cells were isolated using the commercial Dynabeads® Untouched™ Human NK Cell Kit (ThermoFisherScientific/Life Technologies AS, Norway) according to the manufacturer’s recommendations. NK cells were depleted by incubation of freshly isolated PBMCs with 5 μg/ml anti-CD56 (Clone MY31, Cat. No 347740, BD Biosciences, San Jose, CA, USA) and 10 μg/ml LEAF™ purified anti-CD16 (Clone 3G8, Cat. No 302033, Biolegend, San Diego, CA, USA) for 20 minutes on ice and subsequent magnetic separation using Dynabeads® Pan Mouse IgG (ThermoFisherScientific/Life Technologies AS, Norway) according to the manufacturer’s recommendations. Depletion controls are shown in Additional file 1: Figure S1a; NK cells were >90 % depleted; CD56-positive T cells were reduced by this procedure.

### Flow cytometry

PBMCs were stained for 20–30 minutes on ice with a cocktail of monoclonal antibodies, then washed and directly analyzed on a four-laser flow cytometer (LSR Fortessa, BD Biosciences, San Jose, CA, USA). Data were processed using FlowJo® software (FlowJo LCC, Ashland, OR, USA). The following antibodies were used: anti-CD56 Brilliant Violet 421 (Clone NCAM16.2), anti-CD54 PE, anti-CD19 FITC, anti-CD16 FITC (all from BD Biosciences, San Jose, CA, USA); anti-CD3 PE-Dazzle, anti-CD69 PE, anti-CD137 PE (all from Biolegend, San Diego, CA, USA). Where indicated, PBMCs were washed and re-diluted for 20 minutes in Annexin-V (AxV) buffer supplemented with Annexin-V PE before analysis by flow cytometry.

### Degranulation (CD107a) assays

Therapeutic antibodies and anti-CD107a PE-Cy5 (BD Biosciences, San Jose, CA, USA) were added at the same time point to freshly isolated PBMCs (1 × 10^6^ cells/ml). CD107a surface expression on NK cells was measured after culture overnight by flow cytometry as described.

### Cytotoxicity assays

The target cell line K562 was grown in medium (IMDM, 10 % FCS, 1 % penicillin/streptomycin) to mid-log phase: 5 × 10^5^ target cells were labeled in 100 μl assay medium (IMDM with 10 % FCS and penicillin/streptomycin) with 100 μCi of ^51^Cr for 1 to 2 hours at 37 °C. Cells were washed twice in assay medium and resuspended at 5 × 10^4^ cells/ml in assay medium. Five thousand target cells/well were used in the assay. Effector cells (freshly isolated PBMCs) were resuspended in assay medium and mixed with labeled target cells in a V-bottom 96-well plate. Maximum release was determined by incubation in 1 % Triton X-100. For spontaneous release, target cells were incubated without effector cells in assay medium alone. All samples were done in triplicates. Plates were incubated for 4 hours at 37 °C. Supernatant was harvested, and ^51^Cr release was measured in a gamma counter. Percentage of specific release was calculated as:

$$ \left(\left(\mathrm{Experimental}\ \mathrm{release}\ \hbox{--}\ \mathrm{Spontaneous}\ \mathrm{release}\right)/\left(\mathrm{Maximum}\ \mathrm{release}\ \hbox{--}\ \mathrm{Spontaneous}\ \mathrm{release}\right)\right) \times 100. $$

### Statistical analysis

Exploratory statistical analysis was performed. *P* values have to be interpreted descriptively. Normal distribution was not assumed and therefore non-parametric statistical tests were used. The Mann–Whitney test was used to compare two groups. The Wilcoxon signed rank test was used to compare paired samples. All tests were performed with a significance level of 5 % (confidence interval 95 %).

## Results

### Addition of rituximab to PMBCs leads to B cell depletion in the absence of serum

**Fig. 1:**
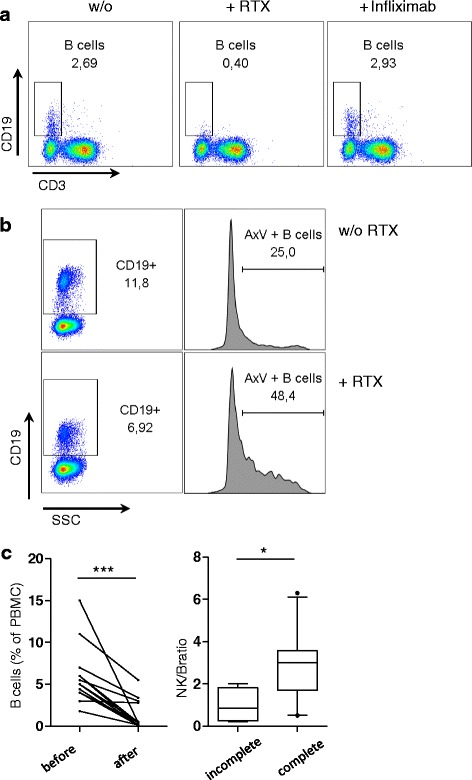
Addition of rituximab to peripheral blood mononuclear cells (*PMBCs*) leads to B cell depletion in the absence of serum. **a** PBMCs were isolated by density gradient centrifugation, left untreated overnight, and cultured over the second night in medium supplemented with heat-inactivated fetal calf serum without any therapeutic antibody (*w/o*, *left panel*), with 10 μg/ml anti-CD20 antibody rituximab (*RTX*) (*middle*) or with 10 μg/ml anti-TNF alpha antibody infliximab (*right panel*). After that, PBMCs were stained with a mixture of lineage antibodies (anti-CD3 PE-Dazzle, anti-CD56 Bv421, anti-CD19 FITC) and analyzed by flow cytometry. B cells were identified as CD3-CD19+ cells in the lymphocyte gate. **b** PBMCs were treated as described (**a**). After washing PBMCs were re-diluted in Annexin-V (AxV) buffer supplemented with PE-labeled Annexin-V and analyzed by flow cytometry. B cells were identified as CD3-CD19+ cells in the lymphocyte gate. *Upper row* culture in medium without RTX (*w/o RTX*). *Lower row* culture in medium with RTX (*+ RTX*). *AxV+ B cells* apoptotic B cells with positive staining for Annexin-V PE. **a**, **b** B cell numbers were reduced after incubation with RTX in 14/14 independent experiments, each performed with PBMCs from different healthy donors. In 10/14 experiments RTX-induced B cell depletion was complete as shown (**a**); in 4/14 B cell depletion was incomplete as shown (**b**). Infliximab was used as a negative control in 2/14 experiments. Increased binding of Annexin-V was identified in three donors with incomplete B cell depletion. **c** *Left graph* B cell percentages before and after culture overnight with rituximab (statistically significant, Wilcoxon signed rank test, *p* = 0.001). *Right graph*, *whiskers* 10–90th percentile. PBMCs with incomplete B cell depletion after incubation with RTX overnight (*incomplete*) had significantly lower ratios of natural killer cells to B cells (*NK/B*) than PBMCSs with complete B cell depletion (*complete*, <0.55 % of PBMCs) (Mann–Whitney *U* test, *p* = 0.03)

Freshly isolated PBMCs from 14 healthy donors were cultured with or without rituximab overnight. In all donors we observed a strong rituximab-mediated reduction in B cell numbers, and no B cells were detectable after rituximab treatment (<0.55 % of lymphocytes) in 10/14 donors (Fig. 1a). In the first experiments, we used anti-TNF alpha antibody infliximab or IvIgs as negative controls. We discontinued these controls in further experiments, as no effects on either the presence of B cells (Fig. 1a; infliximab, *n* = 2; IvIg, *n* = 1) nor the degree of NK cell degranulation was seen (see subsequent text; infliximab, *n* = 3; IvIg, *n* = 2). Infliximab had no effect on B cell proportions, even after culture over 4 days and contrary to rituximab (*n* = 2, not shown). B cell depletion was incomplete in 4/14 donors following rituximab treatment overnight (Fig. 1b, c). The expression of CD19 on the remaining B cells was decreased and viability staining with Annexin V revealed that an important fraction of these cells was apoptotic (Fig. 1b). Donors with incomplete B cell depletion had a significantly lower ratio of NK cells to B cells at baseline than donors with complete B cell depletion (Fig. 1c).

These data indicate that rituximab can induce B cell depletion in PBMCs without the presence of functional serum factors. Low ratios of NK cells to B cells might be responsible for incomplete, presumably delayed, B cell depletion.

### Rituximab leads to NK cell degranulation and downregulation of CD16 in PBMCs

**Fig. 2:**
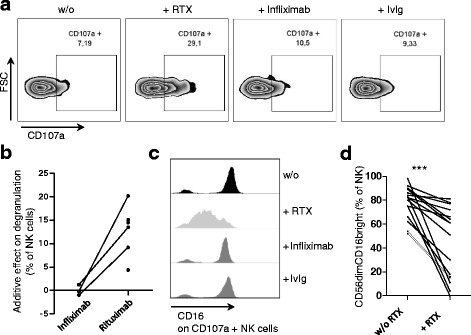
Rituximab (*RTX*) leads to natural killer (*NK*) cell degranulation and downregulation of CD16 in peripheral blood mononuclear cells (PBMCs). PBMCs were isolated and cultured as described in Fig. 1. Anti-CD107a PE-Cy5 was added at the same time point as the therapeutic antibodies (starting point of the degranulation assay). The next day PBMCs were stained with a mixture of antibodies (anti-CD3 PE, anti-CD56 Bv421 and anti-CD16 FITC) and analyzed by flow cytometry. NK cells were identified as CD3-CD56+ cells in the lymphocyte gate. **a** CD107a expression on CD56^dim^ NK cells after stimulation without (*w/o*) therapeutic antibody, with 10 μg/ml RTX (+*RTX*), with 10 μg/ml infliximab and with 10 μg/ml intravenous immunoglobulin (*IvIg*) (from *left* to *right*). Shown is one representative donor. **b** Summary of the increased CD107a expression on total NK cells after treatment with RTX in comparison to infliximab. *Dots linked by a line* belong to the same donor. Additive effect on degranulation is defined by (CD107a pos. NK cells after culture with therapeutic antibody) -(CD107a pos. NK cells after culture without therapeutic antibody). **c** CD16 expression on CD107a-positive NK cells. Shown is one representative donor. **a**-**c** CD107a expression together with CD16 expression has been investigated in healthy individuals (*n* = 6 (+/− RTX), *n* = 3 (+/− infliximab) and *n* = 2 (+/− IvIg)). **d** The percentage of CD16^bright^ cells among CD56^dim^ NK cells before and after stimulation with RTX overnight was investigated in 16 healthy donors. Statistical significance was determined with the Wilcoxon signed rank test (*p* = 0.0005). *FSC* forward scatter

The degranulation of NK cells was measured in six donors after culture of freshly isolated PBMCs with or without rituximab (or control antibody infliximab, *n* = 3, and IvIg, *n* = 2) overnight. CD107a expression as a correlate of degranulation was increased only if rituximab had been added (Fig. 2a, b). In the six donors investigated, CD107a expression was statistically significantly higher in samples that contained rituximab than in samples that contained no therapeutic antibody (Wilcoxon signed rank test, *p* = 0.03; not shown).

The Fc-gamma-receptor CD16 was downregulated on degranulated (CD107a-positive) NK cells, as shown in Fig. 2c. The proportion of CD16^bright^ cells among CD56^dim^ NK cells was determined after culture with or without rituximab in 16 healthy controls. Rituximab led to a significant decrease in CD16^bright^ NK cells (Fig. 2d). The extent of CD16 downregulation varied between donors.

We conclude that rituximab induces NK cell degranulation in healthy PBMCs. Similar to published data in tumor models, rituximab induced downregulation of CD16.

### NK cells and serum cooperate in mediating rituximab-induced B cell depletion

To investigate a causal relationship between NK cell degranulation and the depletion of B cells upon rituximab treatment we depleted NK cells from freshly isolated PBMCs using anti-CD56 and anti-CD16 antibodies and magnetic beads. The remaining PBMCs were cultured overnight with or without rituximab and with or without autologous human serum.

**Fig. 3:**
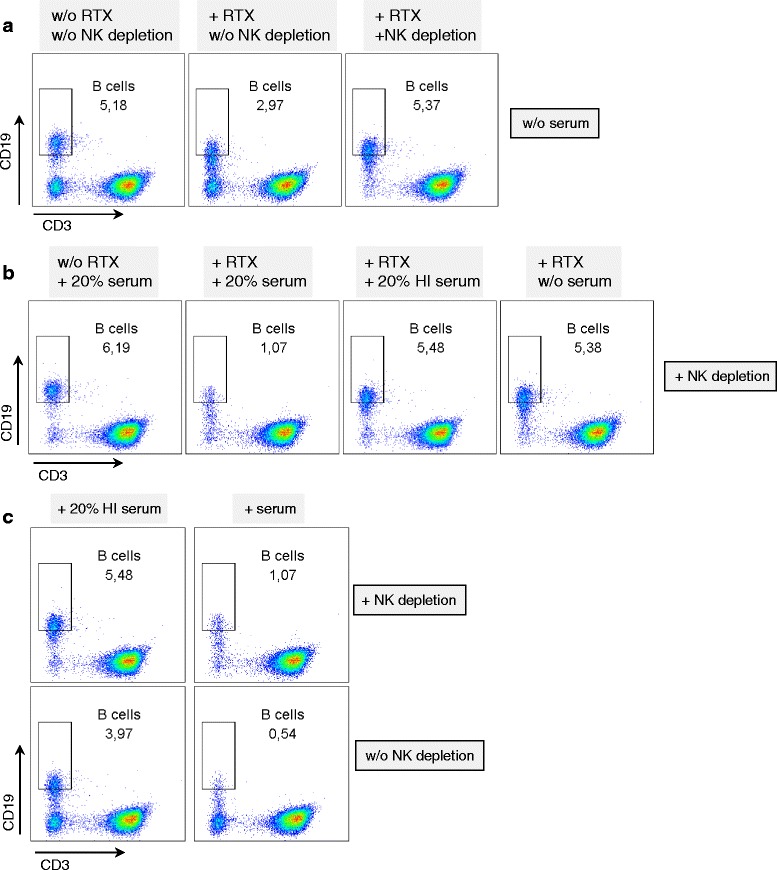
Natural killer (*NK*) cells and serum cooperate in mediating rituximab (*RTX*)-induced B cell depletion. NK cells from freshly isolated peripheral blood mononuclear cells (PBMCs) were depleted (or not) using anti-CD56 and anti-CD16 antibodies and magnetic beads as described in “Methods”. The remaining PBMCs were cultured overnight with (+) or without (*w/o*) RTX and with or without autologous human serum. *HI* heat inactivated. The next day PBMCs were stained and analyzed as described in Fig. 1. **a** All samples were cultured without human serum. **b** NK cells were depleted in all samples. **c** All samples were treated with RTX over night. **a**-**c** Data from the same experiment and donor with incomplete NK cell depletion; the complete experiment with all negative controls including proof of successful NK cell depletion is shown in Additional file 1: Figure S1(a). The effect of NK cell (depletion) and serum on the extent of the B cell depletion and failed RTX-induced B cell depletion in samples without serum and without NK cells were seen in four independent experiments with four different donors

Rituximab-induced B cell depletion was abrogated if NK cells were depleted from the PBMCs (Fig. 3a, *n* = 4 donors). However, the addition of active autologous human serum in addition to rituximab led to reduced numbers of B cells in NK-depleted PBMCs (Fig. 3b, *n* = 4). This effect was abrogated if the serum was heat inactivated, or if serum was not added (Fig. 3b). The greatest reduction in B cells was in samples containing both NK cells and active serum (Fig. 3c). The complete experiment with all negative controls, including the proof of successful NK cell depletion is shown in Additional file 1: Figure S1(a).

These data demonstrate that in the absence of serum, NK cells are responsible for rituximab-induced B cell depletion. In the absence of NK cells, serum factors such as complement alone can mediate rituximab-induced B cell reduction and NK cells and serum factors have a complementary effect on rituximab-induced B cell reduction.

### The extent of rituximab-induced B cell depletion correlates with the size of NK cell proportions

**Fig. 4:**
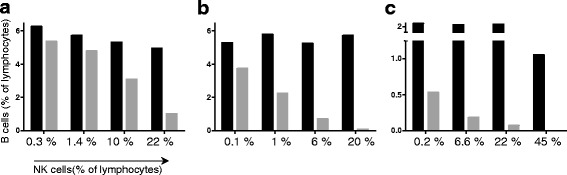
The extent of rituximab-induced B cell depletion correlates with the size of natural killer (*NK*) cell proportions. NK cells were isolated from one fraction of freshly isolated peripheral blood mononuclear cells (PBMCs) and left untreated overnight. Another fraction of freshly isolated PBMCs was left untreated overnight and NK cells were depleted the next day using anti-CD56 and anti-CD16 monoclonal antibodies and magnetic beads. NK-cell-depleted PBMCs were resubstituted with isolated NK cells in three different proportions. The samples were incubated with (*gray bars*) or without (*black bars*) 10 μg/ml rituximab overnight. PBMCs were stained and analyzed by flow cytometry as described in Fig. 1. **a**-**c** Three independent experiments. *First bars* show NK cell depletion without re-substitution. *Second to fourth bars* show NK cell depletion with resubstitution of different percentages of NK cells as indicated. **a** Data from the same experiment shown in Fig. 3 (cultured without human serum); the complete experiment with all negative controls including proof of successful NK cell depletion is shown in Additional file 1: Figure S1(b). **b**, **c** Two further experiments performed with different donors (cultured with autologous human serum). The statistical analysis is described in “Results”

To investigate the correlation between the amount of B cell depletion and the ratio of NK to B cells (Fig. 1), we resubstituted NK-cell-depleted PBMCs with autologous NK cells from three different healthy donors (Fig. 4a, b, and c, respectively). The addition of increasing amounts of NK cells led to increasingly profound decrease in B cells (Fig. 4, gray bars). This decrease was not an indirect consequence of increased NK cell proportions, as shown by the negative control samples cultured without rituximab (Fig. 4, black bars). One complete experiment with all combination scenarios (+/− rituximab, +/− NK cell depletion, +/− NK cell re-substitution) is shown in Additional file 1: Figure S1(b).

In order to statistically analyze this effect, the ratio (B cells (% of lymphocytes) in samples treated with rituximab)/(B cells in samples treated without rituximab) was calculated and related to the respective NK cell percentages (% of lymphocytes). The linear regression analysis of the donor shown in Fig. 4a was significant (*R*^2^ = 0.9993, *p* = 0.0003), whereas the analysis of the donors shown in Fig. 4b and c were not significant (most likely due to limited measurements per donor). The pooled correlation analysis was significant (Spearman's *r* = −0.736, *p* = 0.0047). We conclude that B cell depletion can be accelerated by elevated NK cell proportions.

### Rituximab leads to further phenotypic changes in CD56^dim^ NK-cells

**Fig. 5:**
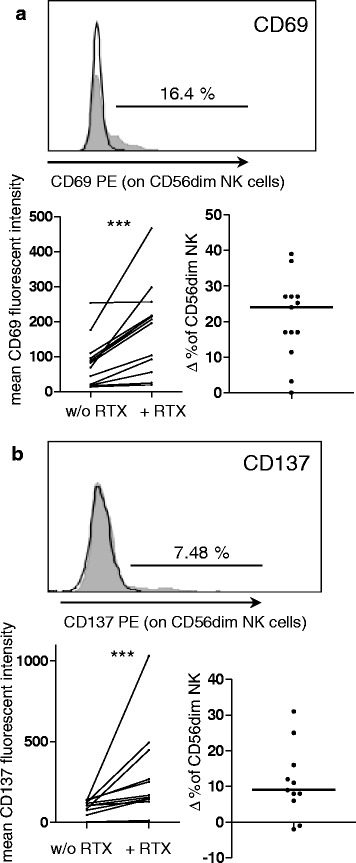
Rituximab (*RTX*) leads to further phenotypical changes in CD56^dim^ natural killer (*NK*) cells. Freshly isolated peripheral blood mononuclear cells (PBMCs) were incubated with (+) or without (*w/o*) RTX overnight. The next day PBMCs were stained with a mixture of antibodies (anti-CD3 PE-Dazzle, anti-CD56 Bv421 and anti-CD16 FITC and CD69/CD137 PE) and analyzed by flow cytometry. NK cells were identified as CD3-CD56+ cells in the lymphocyte gate and fluorescence intensity of PE on CD56^dim^ NK cells was measured. **a** CD69 PE. *Histogram* shows one representative donor; *gray* incubation + RTX, *black* w/o RTX. *Left diagram* mean CD69 PE fluorescence intensity in samples from 13 donors. Statistical *significance was determined with the Wilcoxon signed rank test (p = 0.0002). Right diagram* Δ % in CD56^dim^ NK was determined by (CD69-positive CD56^dim^ NK cells after incubation + RTX) - (CD69-positive CD56^dim^ NK cells after incubation w/o RTX); *bar* median. **b** CD137(41BB)-PE; same donor, layout and abbreviations as in **a**; *n* = 11 donors; *p* = 0.001

We investigated the expression of the lymphocyte activation marker CD69 on CD56^dim^ NK cells from freshly isolated PBMCs from 13 healthy donors. After culture with rituximab overnight, CD69 expression was statistically significantly greater than after culture without rituximab (Wilcoxon signed rank test, *p* = 0.0002; Fig. 5a). A median of 25 % of CD56^dim^ NK cells expressed CD69 de novo.

Using the same culture conditions, the expression levels of the NK cell co-activation receptor CD137 (41BB) was investigated in 11 donors (Fig. 5b). Rituximab significantly increased the expression of CD137 (*p* = 0.001). A median of about 10 % of CD56^dim^ NK cells expressed CD137 as a response to rituximab.

Therefore, next to the effects of rituximab that can be directly assigned to Fc-γ-receptor CD16 signaling (such as degranulation and downregulation of CD16), an important fraction of CD56^dim^ NK cells showed unspecific signs of activation and the expression of at least one co-activating receptor was altered.

### NK cells exposed to rituximab can become hypo-responsive in a donor-dependent fashion

We hypothesized that the phenotypic changes described might impact the NK cell cytotoxicity towards other target cells. To explore this, we performed a series of killing assays with 11 healthy donors. Freshly isolated PBMCs were cultured overnight with or without rituximab, washed, and then co-cultured for 4 hours with the NK cell-sensitive, ^51^Cr-labeled K562 cell line. Lysis of K562 cells by NK cells is independent of Fc-receptors.

**Fig. 6:**
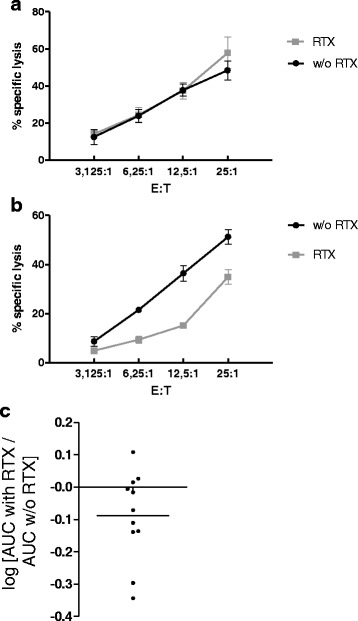
Natural killer (NK) cells exposed to rituximab (*RTX*) can become hypo-responsive in a donor-dependent fashion. Freshly isolated peripheral blood mononuclear cells (PBMCs) from 11 healthy donors were cultured overnight with or without (*w/o*) RTX in medium without human serum. After washing, PBMCs (effector cells (*E*)) and ^51^Cr-labeled K562 (target (*T*)) cells were co-cultured for 4 hours. Percent specific lysis was determined by measuring ^51^Cr in the supernatant. For every donor a ratio of the % specific lysis with and without RTX ((RTX)/(w/o RTX)) was calculated for each E/T and statistically analyzed by comparison with a hypothetic mean value of 1 using the one-sample *t* test. **a** Although eight donors had no significant changes in NK cell cytotoxicity (**b**) we observed that RTX significantly inhibited NK cell cytotoxicity in three donors (*p* < 0.05). **c** To compare all donors, we calculated the area under the curve (*AUC*) for each cytotoxicity assay with and without RTX pretreatment. Shown is log10 (AUC with RTX)/(AUC w/o RTX). Data were analyzed using the one-sample *t* test (not significant, *p* = 0.0593; *bar* mean)

We observed donor-dependent effects of rituximab on NK cell cytotoxicity. While many donors had no reduction or only slight reduction in cytotoxicity after culture with rituximab (Fig. 6a) we observed statistically significant reduction in NK cell cytotoxicity upon rituximab pretreatment in three donors (Fig. 6b). Due to this donor variability, on statistical analysis of the overall NK cell cytotoxicity in all donors there was a non-significant trend towards reduced NK cell cytotoxicity upon culture with rituximab (Fig. 6c).

These data indicate that incubation of non-malignant PBMCs with rituximab alters NK cell cytotoxicity in a donor-dependent fashion.

## Discussion

Anti-CD20 tumor therapy with rituximab has broad applications in hematology. Many studies confirm its clinical and mechanistic effects, and NK cells are a well-described mediator of rituximab-induced tumor lysis [21]. The progress in understanding rituximab-induced NK cell-mediated effects has recently been highlighted by the finding that NK cells are less cytotoxic after co-culture with rituximab-coated tumor cells [7]. Indeed, multiple activating pathways are blocked through internalized CD16 that atypically binds the phosphatase SHP1, which in turn leads to the disruption of central steps of NK cell activation [7]. This finding is of the upmost importance in lymphoma therapy. Lymphomas often consist of CD20-positive and CD20-negative transformed B cells: although CD20-positive cells could be successfully defeated with rituximab, the CD20-negative fraction could be less successfully suppressed by NK cells with reduced cytotoxicity, and grow out, leading to rituximab resistance and a worse outcome. Apart from the findings discussed subsequently, the present study is the first to confirm inhibited NK cell cytotoxicity after pretreatment with rituximab in a non-malignant experimental setting.

Rituximab also has broad and increasing applications in rheumatology. However, there is a lack of mechanistic studies. Although B cell therapy is believed to be central in mediating the anti-inflammatory effect of rituximab, several clinical findings cannot be explained by it or seem to be contradictory. For example, despite the initial idea that depletion of B cells would diminish pathogenic autoantibodies, elevated serum levels of autoantibodies do not strictly correlate with clinical response [26], and can persist despite effective rituximab treatment. Vice versa, treatment response can fail, even with successful B cell depletion. Essentially and due to the absence of biomarkers, it remains unclear, which patients would benefit from rituximab and which would be unresponsive to it.

The transfer of knowledge from cancer studies to rheumatic disease might be erratic because of secondary effects intrinsic to tumor cells [19]. Therefore, studies of rituximab under non-malignant conditions are needed to identify the precise conditions that are required for successful treatment of rheumatic diseases with rituximab, and to understand the previously mentioned clinical discrepancies. The present study was supposed to provide a fundamental “health condition” basis for further studies on the interactions between rituximab and NK cells in rheumatic disease.

Our first major finding is the participation of NK cells in mediating rituximab-induced B cell depletion in our non-malignant experimental setting. This finding is supported by the association between incomplete or delayed B cell depletion and a low ratio of NK cells to B cells, by the specifically increased degranulation and the downregulation of CD16, by the absence of B cell depletion in the case of concomitant NK cell depletion (and missing serum factors), by the correlation between the extent of B cell reduction and the amount of re-substituted NK cells and finally, by distinct phenotypic changes in rituximab-treated samples.

NK cells and serum (presumably complement) factors seem to synergize. This is interesting, as both parameters can be deficient in diverse clinical situations. It would be interesting to investigate whether NK cells play a more prominent role in hypocomplementemia, e.g. in active systemic lupus erythematosus; or whether complement-dependent effects are more pronounced in situations where NK cell numbers are reduced, e.g. in highly active GPA (own data, publication in preparation). The two parameters might also be differently relevant depending on the location. NK cells are not detectable in GPA granulomas [27], but B cells are present [28]. Herein may lay the reason for the limited efficacy of rituximab in treating granulomatous lesions, and why B cells can be found in granulomas despite complete depletion in peripheral blood [29]. In any case, NK cells are mediators of rituximab-induced B cell depletion in a non-malignant setting. We suggest that NK cell proportions in peripheral blood and tissue lesions should be systematically investigated in rheumatic disease in the context of rituximab therapy.

Direct apoptotic effects of rituximab or ADCC by monocytes do not seem to be relevant in our setup. In line with this, in PBMCs NK cells, but not monocytes, have been found to mediate cytotoxicity towards rituximab-coated tumor cells [30]. However, a marginal direct apoptotic effect might not be completely excluded and could explain the slight shift in the B cell population from Fig. 3a (left and right graph).

The second major finding of the present study is the altered NK cell function after pretreatment with rituximab. It must be noted that the only other study that described NK cell inhibition by rituximab was performed using large amounts of rituximab-coated tumor cell line cells [7]. Thus, this study investigated rituximab-induced NK cell changes after a maximum of a stimulus. Our experimental ex vivo setting, on the contrary, operates in the most physiological fashion by using PBMCs. Essentially, the ratios of NK cells to target cells (B cells) were unaltered, and effects on NK cells are likely to be transferable in vivo. Therefore, it would be interesting to investigate whether the inhibited NK cell cytotoxicity might be relevant in the treatment of patients with non-malignant disease.

This raises the question about a possible consequence of decreased NK cell cytotoxicity in rheumatic diseases. We can only speculate about this. Decreased NK cell cytotoxicity by rituximab could lead to a clinical benefit if NK cells are pathogenic, e.g. hyperactive in a given disease. On the other hand, it could have a detrimental effect if NK cells have a protective role. So far, the involvement of the function of NK cells is not established in most rheumatic diseases, but they could play a role, e.g. in Sjögren’s syndrome or GPA [27, 31].

We could not make up differences between donors with decreased and unaltered cytotoxicity after treatment with rituximab. The ratio of NK cells to B cells and loss of CD16^bright^ cells were not different between the groups (not shown), but the subgroups were too small to draw any conclusions. The factors causing decreased cytotoxicity after rituximab-treatment remain unknown. Possible reasons could comprise CD16 polymorphisms with different affinity to rituximab, counter-regulation by unknown factors (see also subsequent text), time dependence and sensitivity of our experimental setup, or others. Clinical trials should investigate whether there is correlation between altered NK cell cytotoxicity and response to rituximab treatment.

To summarize, these data show that rituximab induces altered NK cell phenotype and function, and the clinical significance of this needs to be established. These data are fundamental in understanding how rituximab works in non-malignant diseases, and will lead to further studies investigating the role of NK cells and rituximab in rheumatic diseases.

Another important finding is the upregulation of the co-activating receptor CD137 (41BB) on a subset of CD56^dim^ NK cells. This finding is precious, as all other activating receptors investigated so far are not altered after rituximab pretreatment (NKG2D, 2B4, NKp46, NKp30, DNAM1, LFA1 and CD2-receptor) [7]. Upregulation of this receptor could be a reason why the reduction of NK cell cytotoxicity is relatively weak, as the tumor cell line K562 expresses small amounts of CD137 ligand (CD137L, 41BBL, not shown). Therefore, the results from Fig. 6 could underestimate the extent of the reduction in killing, depending on the nature of a given target cell. The finding of upregulation of CD137 could be of clinical importance, as agonist anti-CD137 antibodies are tested for clinical use [21]. The idea is to combine a tumor binding antibody with agonist anti-CD137 antibody in order to enhance NK cell cytotoxicity towards tumor cells [8, 32]. We suggest that such a combination might also be applicable in rheumatic disease, e.g. in patients with incomplete B cell depletion or response to rituximab. Furthermore, anti-CD137 can be used to enhance NK cell proliferation in vitro [33, 34], and NK cell numbers had been shown to be inversely correlated with GPA activity [27]. It would be interesting to test agonist anti-CD137 antibody in combination with rituximab in rheumatic diseases.

## Conclusions

In summary, we show that NK cells mediate rituximab-induced B cell depletion in a non-malignant experimental setting. Rituximab induces altered NK cell phenotype and function, for which the clinical significance needs to be investigated.

## Additional file

Additional file 1:
**Figure S1a.** NK cells and serum cooperate in mediating rituximab-induced B cell depletion. Same experiment as shown in Fig. 3; all samples and controls are shown. -/+ RTX, without/with rituximab. **Figure S1b.** The extent of rituximab-induced B cell depletion correlates with the size of NK cell proportions. Same experiment as shown in Fig. 4a; the complete experiment with all negative controls including proof of successfull NK cell depletion is shown. -/+ RTX, without/with rituximab. (ZIP 421 kb)

## Abbreviations

2B4: CD244
41BB: CD137
^51^Cr: Chrome-51
ADCC: Antibody-dependent cellular cytotoxicity
CD: Cluster of differentiation
CD137L: CD137 ligand
DNAM1: CD226, DNAX accessory molecule-1
FCS: Fetal calf serum
GPA: Granulomatosis with polyangiitis
IgG: Immunoglobulin G
IvIgs: Intravenous immunoglobulins
LFA1: Lymphocyte function-associated antigen 1 (CD11a/CD18 Integrine)
mAb: Monoclonal antibody
NK cell: Natural killer cell
NKG2D: Natural-killer group 2, member D; activating NK-cell receptor
NKp30/NKp46: Natural killer cell p30/46-related protein.
PBL: Peripheral blood lymphocyte
PBMC: Peripheral blood mononuclear cell
RTX: Rituximab
SHP1: Src homology region 2 domain-containing phosphatase-1
TNF: Tumor necrosis factor
w/o: Without
