# Supplementary material for: Rituximab induces phenotypical and functional changes of NK cells in a non-malignant experimental setting
Source: Arthritis Res Ther. 2016 Sep 15;18:206. doi: 10.1186/s13075-016-1101-3 (PMC5024429; doi:10.1186/s13075-016-1101-3)
Supplement: Additional file 1: — Figure S1a. NK cells and serum cooperate in mediating rituximab-induced B cell depletion. Same experiment as shown in Fig. 3; all samples and controls are shown. -/+ RTX, without/with rituximab. Figure S1b. The extent of rituximab-induced B cell depletion correlates with the size of NK cell proportions. Same experiment as shown in Fig. 4a; the complete experiment with all negative controls including proof of successfull NK cell depletion is shown. -/+ RTX, without/with rituximab. (ZIP 421 kb) [file 13075_2016_1101_MOESM1_ESM.zip › wm 2016 02 11 Figure S1a.pdf]

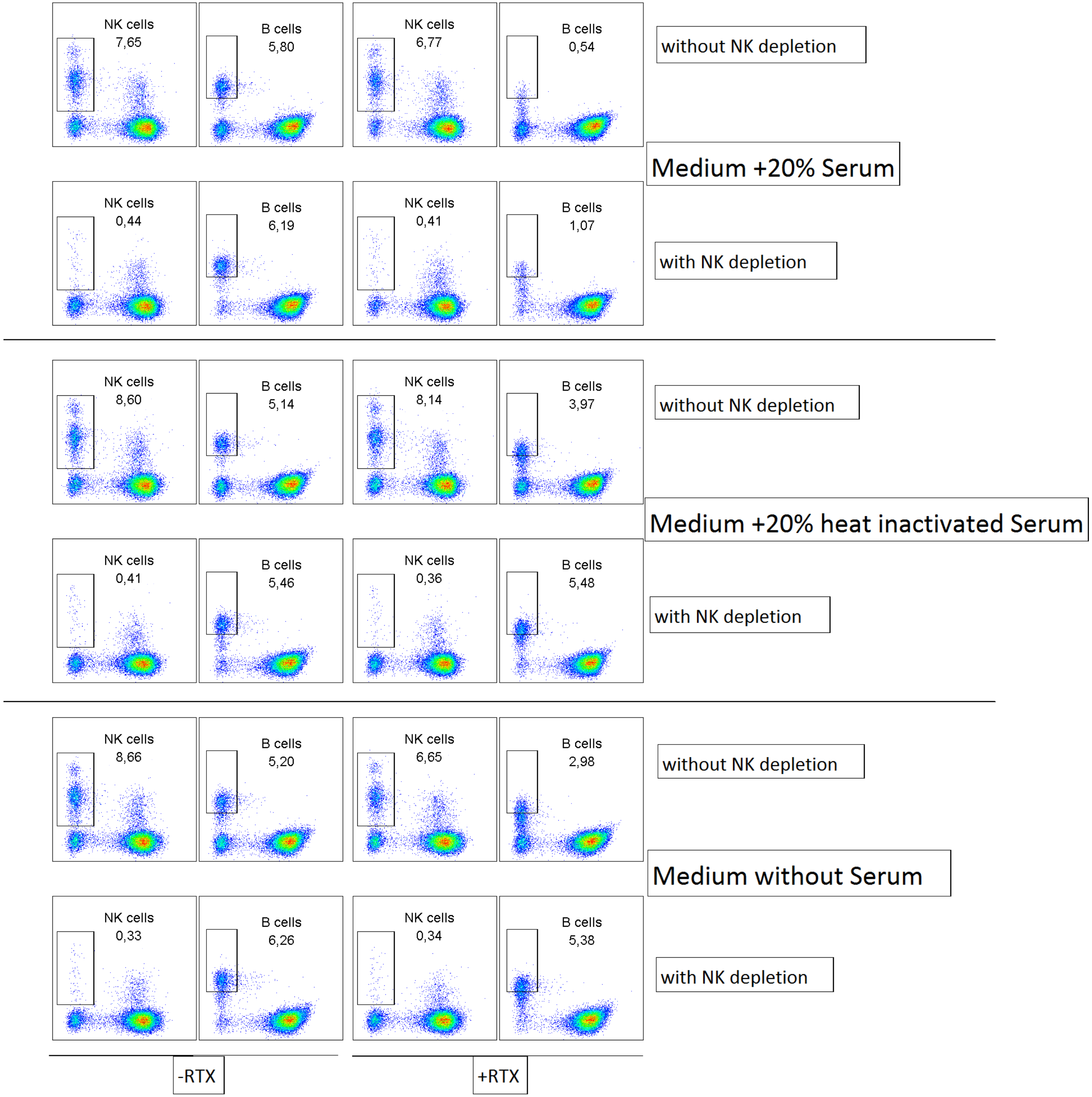
NK cells 7,65
B cells 5,80
NK cells 6,77
B cells 0,54
without NK depletion
Medium +20% Serum
NK cells 0,44
B cells 6,19
NK cells 0,41
B cells 1,07
with NK depletion
NK cells 8,60
B cells 5,14
NK cells 8,14
B cells 3,97
without NK depletion
Medium +20% heat inactivated Serum
NK cells 0,41
B cells 5,46
NK cells 0,36
B cells 5,48
with NK depletion
NK cells 8,66
B cells 5,20
NK cells 6,65
B cells 2,98
without NK depletion
Medium without Serum
NK cells 0,33
B cells 6,26
NK cells 0,34
B cells 5,38
with NK depletion
-RTX
+RTX

S1a
