# Supplementary figures and images for: Rituximab induces phenotypical and functional changes of NK cells in a non-malignant experimental setting
Source: Arthritis Res Ther. 2016 Sep 15;18:206. doi: 10.1186/s13075-016-1101-3 (PMC5024429; doi:10.1186/s13075-016-1101-3)

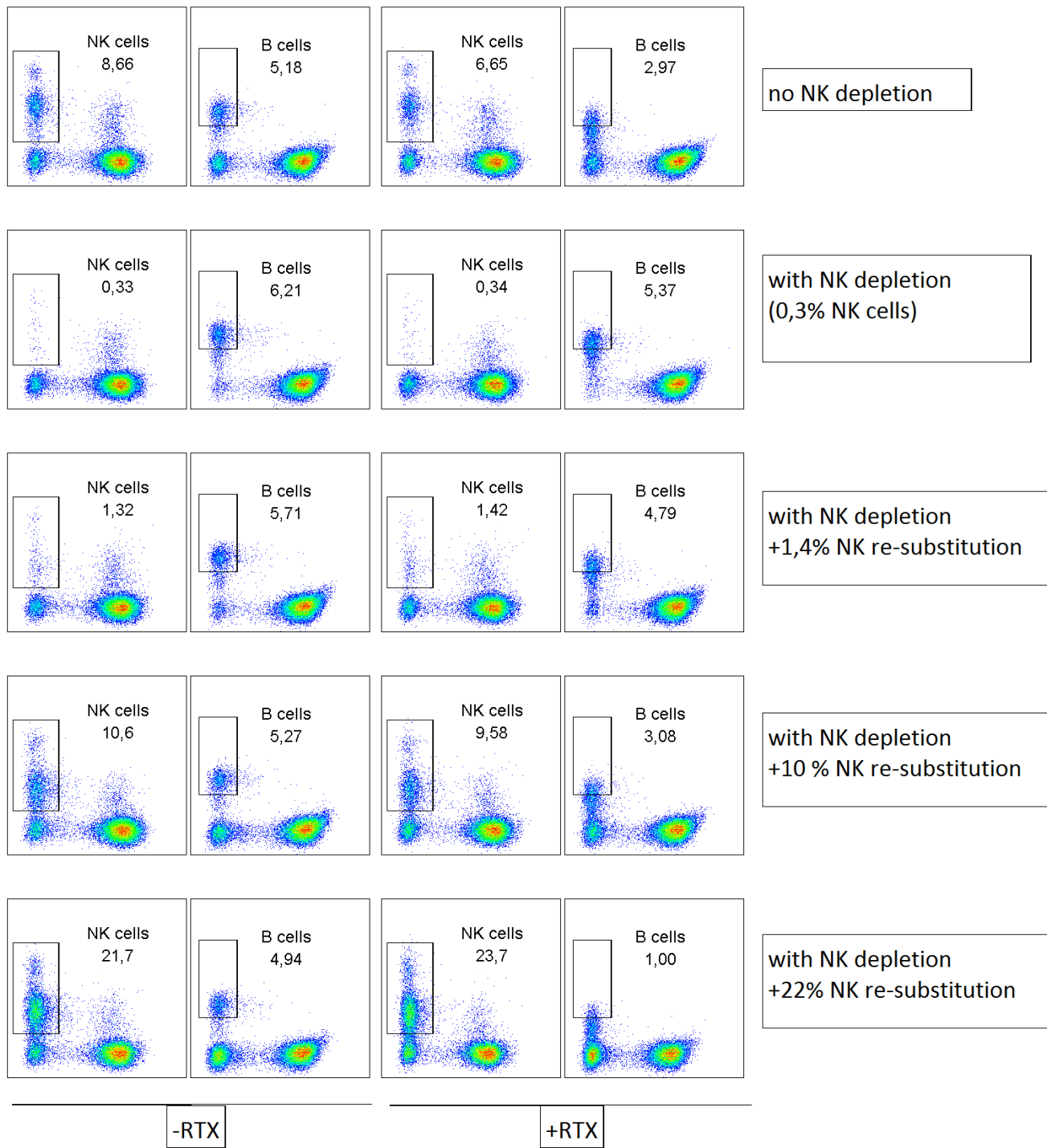

Supplement: Additional file 1: — Figure S1a. NK cells and serum cooperate in mediating rituximab-induced B cell depletion. Same experiment as shown in Fig. 3; all samples and controls are shown. -/+ RTX, without/with rituximab. Figure S1b. The extent of rituximab-induced B cell depletion correlates with the size of NK cell proportions. Same experiment as shown in Fig. 4a; the complete experiment with all negative controls including proof of successfull NK cell depletion is shown. -/+ RTX, without/with rituximab. (ZIP 421 kb) [file 13075_2016_1101_MOESM1_ESM.zip › wm 2016 02 11 Figure S1b.pdf]
